# Pott's Spine Presenting With Concurrent Paraspinal, Retropharyngeal, and Wrist Abscesses: A Rare Case Report

**DOI:** 10.1002/ccr3.73445

**Published:** 2026-08-30

**Authors:** Bibek Shrestha, Sangam Neupane, Nikhil Ranjan Das, Subodh Acharya, Santosh Khadka, Shrawan Dhungana, Dinesh Kafle

**Affiliations:** ^1^ Maharajgunj Medical Campus Tribhuvan University Institute of Medicine Kathmandu Nepal; ^2^ Department of Orthopedics and Trauma Surgery Tribhuvan University Teaching Hospital Kathmandu Nepal

**Keywords:** abscess, retropharyngeal abscess, spinal tuberculosis, wrist abscess

## Abstract

Spinal tuberculosis may present atypically with multifocal cold abscesses without neurological deficit or constitutional symptoms. Concurrent lumbosacral paraspinal, retropharyngeal, and wrist abscesses, though rare, should raise suspicion for tuberculosis, and early diagnosis with MRI and GeneXpert enables effective minimally invasive management with anti‐tubercular therapy.

## 1 Introduction

Spinal tuberculosis (Pott's spine) is the most common form of skeletal tuberculosis, accounting for nearly half of all cases of musculoskeletal TB [1]. It typically involves the thoracolumbar region and may lead to vertebral destruction, deformity, and neurological deficits if not diagnosed and treated early [2].

A characteristic feature of spinal tuberculosis is the formation of cold abscesses, which may extend along fascial planes to distant sites [3]. Paravertebral and psoas abscesses are commonly reported; however, simultaneous involvement of anatomically distant regions, such as the lumbosacral paraspinal area and retropharyngeal space, is rare [4]. Retropharyngeal abscesses are more frequently associated with cervical spine involvement and can present with airway compromise, dysphagia, or neck swelling, making their occurrence alongside lower spinal disease unusual [5].

Here, we report a rare case of Pott's spine presenting with concurrent lumbosacral paraspinal, retropharyngeal, and wrist abscesses in a young adult without neurological deficit. Such multifocal involvement at anatomically distant sites is exceedingly uncommon and highlights the potential for hematogenous dissemination in tuberculosis. This case underscores the importance of maintaining a high index of suspicion for tuberculosis in patients presenting with multiple cold abscesses and atypical clinical features, enabling timely diagnosis and effective management.

## 2 Case History/Examination

A 31‐year‐old male presented with complaints of pain and swelling over the left paralumbar region for 4 months, associated with swelling over the right side of the neck for 1 month and right wrist pain for 1 month. There was no history of trauma, fever, or constitutional symptoms. Bowel and bladder habits were normal. There was no previous history of pulmonary or extrapulmonary tuberculosis, prior anti‐tubercular treatment, or known close contact with a patient with tuberculosis.

On physical examination, the patient was afebrile with stable vital signs and fair general condition. Local examination revealed a diffuse, nontender swelling measuring approximately 10 × 10 cm over the left paraspinal lumbar region. There was no spinal tenderness. Straight leg raising test (SLRT) was 0°–80° on the right and 0°–90° on the left. Neurological examination of the lower limbs showed preserved motor function (L2–S1: 5/5 power bilaterally), intact sensation, normal knee and ankle jerks, and bilaterally downgoing plantar reflexes.

## 3 Methods (Differential Diagnosis, Investigations and Treatment)

**FIGURE 1:**
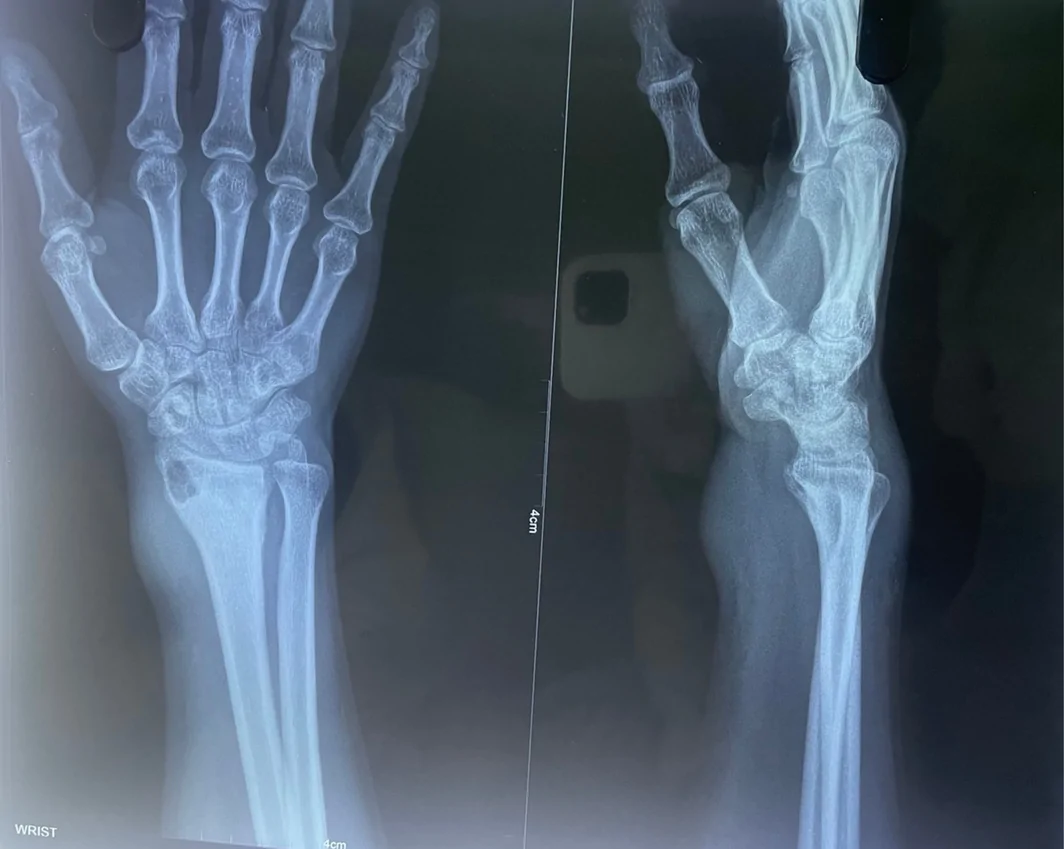
Plain radiograph of the right wrist demonstrating diffuse soft‐tissue swelling without obvious osseous destruction.

**FIGURE 2:**
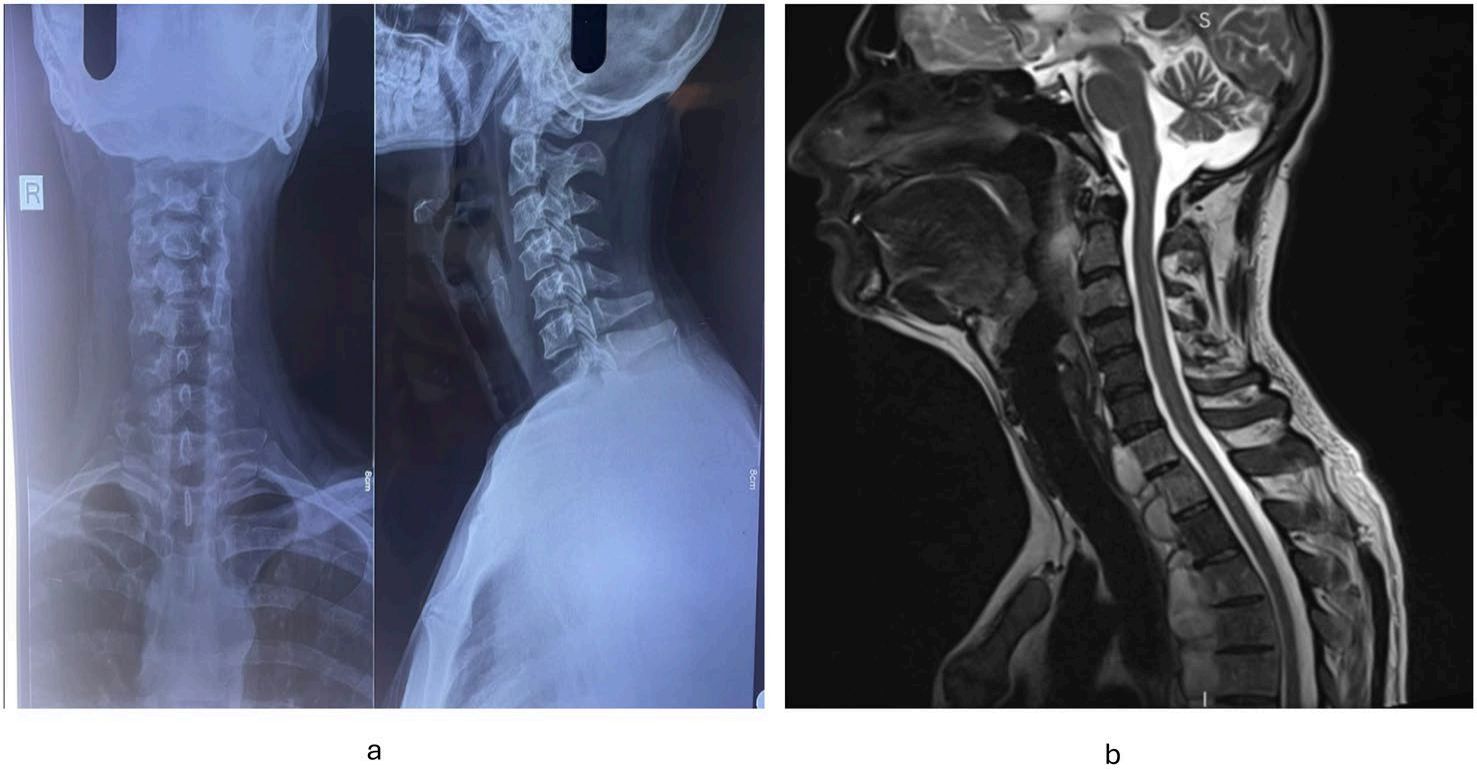
(a) Plain radiograph of the cervical spine (AP and lateral views) showing increased prevertebral soft tissue shadow on the lateral view, suggestive of retropharyngeal soft tissue swelling. (b) Sagittal T2‐weighted MRI of the cervical spine showing a hyperintense prevertebral collection consistent with retropharyngeal abscess, causing anterior displacement of the posterior pharyngeal wall.

Laboratory investigations revealed hemoglobin of 10 g/dL, total leukocyte count of 6600/mm^3^, and platelet count of 335,000/mm^3^. Serology was nonreactive. Ultrasound‐guided aspiration of the left lumbosacral paraspinal collection was performed, and the aspirated pus was sent for GeneXpert MTB/RIF testing, which detected *Mycobacterium tuberculosis* . The patient also complained of right wrist pain for 1 month; further evaluation revealed a wrist abscess on radiographic imaging, which was subsequently drained (Figure 1). Plain radiograph of the cervical spine (anteroposterior and lateral views) demonstrated increased prevertebral soft tissue thickness on the lateral view, suggestive of prevertebral soft tissue swelling, without obvious vertebral body collapse or malalignment (Figure 2a).

**FIGURE 3:**
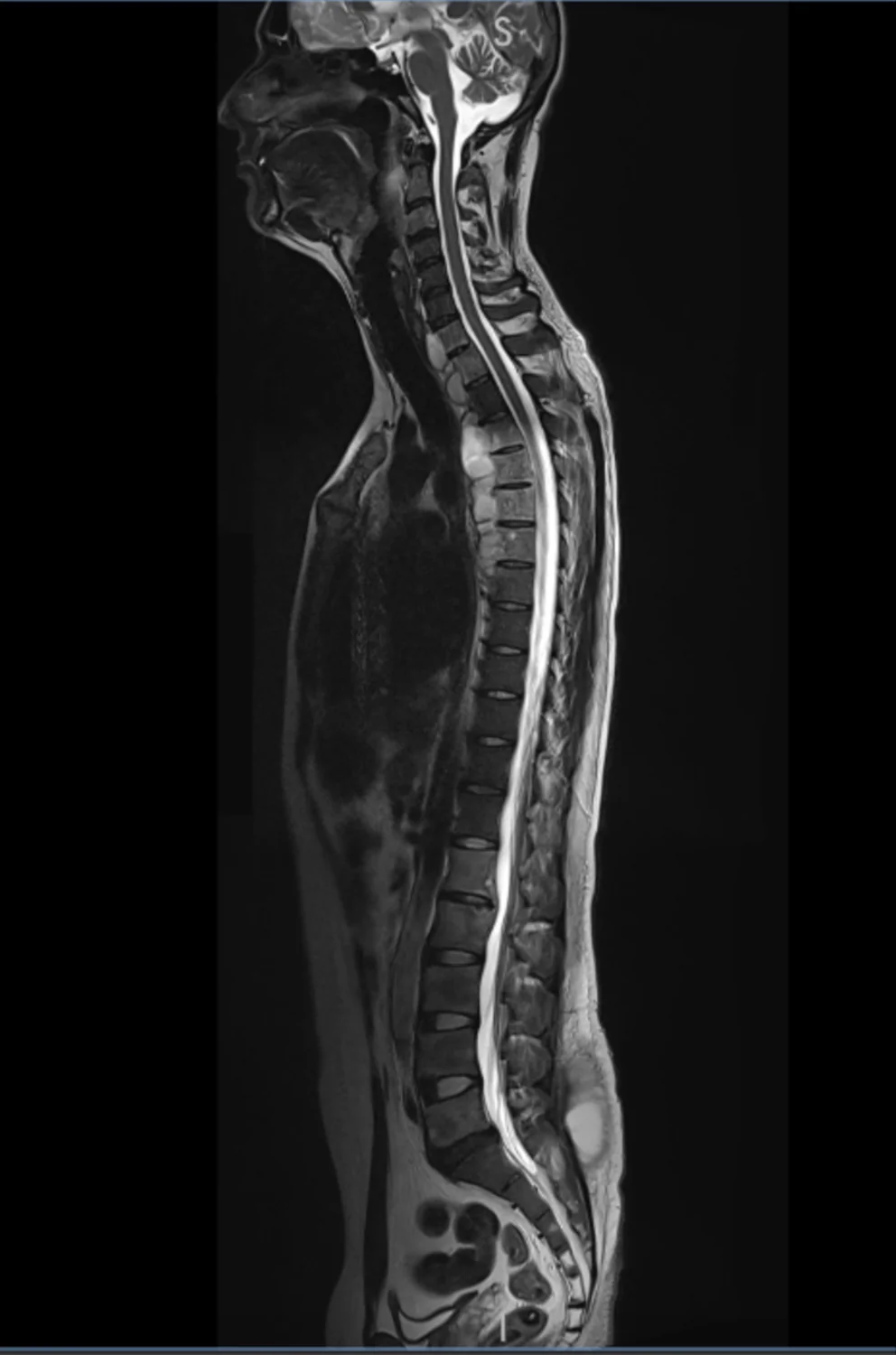
Sagittal T2‐weighted MRI of the whole spine showing hyperintense paraspinal collection in the lumbosacral region consistent with cold abscess, with associated vertebral involvement suggestive of tuberculous spondylodiscitis.

Magnetic resonance imaging (MRI) of the cervical spine (sagittal T2‐weighted sequence) revealed a well‐defined hyperintense collection in the prevertebral region extending along the cervical vertebral levels, consistent with a retropharyngeal abscess. The lesion caused anterior displacement of the posterior pharyngeal wall, with no evidence of spinal cord compression or intramedullary signal changes (Figure 2b). Further evaluation with whole‐spine MRI demonstrated features of infective spondylodiscitis involving the lower lumbar spine, with an associated large hyperintense paraspinal collection in the lumbosacral region, consistent with a cold abscess. No significant spinal canal compromise was noted (Figure 3). Based on clinical, radiological, and microbiological findings, a diagnosis of Pott's spine (spinal tuberculosis) with left lumbosacral paraspinal abscess and retropharyngeal abscess was made.

The patient underwent pigtail catheterization for drainage of the lumbosacral abscess under appropriate anesthesia. Anti‐tubercular therapy (ATT) was initiated with a 12‐month regimen consisting of a 2‐month intensive phase with isoniazid, rifampicin, pyrazinamide, and ethambutol (2HRZE), followed by a 10‐month continuation phase with isoniazid, rifampicin, and ethambutol (10HRE). During hospitalization, the patient showed clinical improvement with reduction in swelling and stabilization of symptoms.

## 4 Conclusions and Results (Outcome and Follow‐Up)

At discharge, the patient was hemodynamically stable, with a healthy wound and no neurological deficits. He was advised to continue anti‐tubercular therapy along with supportive medications including antibiotics, proton pump inhibitors, pyridoxine, and neuropathic pain agents. At follow‐up, the patient demonstrated satisfactory clinical improvement. The paraspinal and neck swellings had markedly reduced, with resolution of pain and improvement in right wrist symptoms. He remained afebrile and had no constitutional symptoms, neurological deficits, bowel or bladder disturbances, dysphagia, or respiratory complaints. The patient was ambulatory and able to perform his routine activities without significant limitation. At the latest clinical follow‐up, there was no recurrence or worsening of the swellings or development of new neurological symptoms.

## 5 Discussion

Spinal tuberculosis remains a major global health concern, particularly in endemic regions, and continues to pose diagnostic challenges due to its variable and often indolent presentation [6, 7]. Although the thoracolumbar spine is most frequently involved, the disease spectrum is broad, ranging from isolated vertebral involvement to extensive soft tissue abscess formation and neurological compromise [8]. In the present case, the coexistence of lumbosacral paraspinal abscess and retropharyngeal abscess represents an unusual and clinically significant manifestation.

The formation of cold abscesses in spinal tuberculosis occurs due to caseous necrosis and liquefaction, allowing pus to track along tissue planes with minimal inflammatory response [9]. While paravertebral and psoas abscesses are commonly described, the presence of a retropharyngeal abscess is typically associated with cervical spine involvement rather than lower spinal disease [10]. The occurrence of both lumbosacral, retropharyngeal and wrist abscesses in this patient suggests the possibility of hematogenous dissemination or contiguous spread along fascial planes, highlighting the multifocal nature of tuberculosis infection. Similar cases have been reported rarely in the literature, underscoring the atypical distribution seen in this patient [11, 12].

Another noteworthy feature in this case is the absence of constitutional symptoms and neurological deficits despite significant disease burden. Classically, spinal tuberculosis presents with back pain, weight loss, fever, and, in advanced stages, neurological involvement due to spinal cord compression [13]. However, several studies have emphasized that early or atypical cases may lack these hallmark features, leading to delays in diagnosis [4, 8, 10]. The preserved neurological status in our patient, despite the presence of sizeable abscesses, may be attributed to the slow progression of the disease and lack of significant spinal canal compromise.

The diagnostic approach in this case highlights the importance of microbiological confirmation. GeneXpert testing of aspirated pus provided rapid and reliable identification of *Mycobacterium tuberculosis* , facilitating early initiation of anti‐tubercular therapy [14]. In resource‐limited settings, such molecular techniques are increasingly valuable, especially in atypical presentations where clinical suspicion may be low. Imaging modalities, particularly MRI, play a crucial role in delineating the extent of disease and identifying associated abscesses, although microbiological confirmation remains the gold standard [1].

Management of spinal tuberculosis is primarily medical, with anti‐tubercular therapy forming the cornerstone of treatment [15]. However, intervention is often required in cases with large abscesses, neurological deficits, or diagnostic uncertainty [1, 16]. In this patient, minimally invasive pigtail catheter drainage of the lumbosacral abscess, combined with standard ATT, resulted in favorable clinical outcomes. The presence of a retropharyngeal abscess in spinal tuberculosis warrants particular attention due to the potential risk of airway compromise and rapid clinical deterioration [11, 12]. Although our patient did not exhibit airway symptoms, early recognition and close monitoring are essential. This case therefore emphasizes the need for comprehensive evaluation in patients with spinal tuberculosis, including assessment for distant or atypical abscess formation.

## Author Contributions

**Nikhil Ranjan Das:** supervision, validation. **Sangam Neupane:** supervision, validation. **Subodh Acharya:** supervision, validation. **Santosh Khadka:** supervision, validation. **Dinesh Kafle:** supervision, validation. **Shrawan Dhungana:** supervision, validation. **Bibek Shrestha:** conceptualization, writing – review and editing, writing – original draft, visualization.

## Funding

The authors have nothing to report.

## Ethics Statement

Ethical approval was not required for this case report in accordance with local, regional, and national guidelines.

## Consent

Written informed consent was obtained from the patient for publication of this case report and accompanying images.

## Conflicts of Interest

The authors declare no conflicts of interest.

## Data Availability

The data supporting the findings of this study are available from the corresponding author upon reasonable request, with patient confidentiality maintained.
